# The Treatment of Fractures

**Published:** 1902-12

**Authors:** 


The Treatment of Fractures. By Chas. L. Scudder, M. D., Assistant in Clinical and Operative Surgery, Harvard Medical School. Third edition, revised and enlarged. Octavo, 480 pages, with 645 original illustrations. Philadelphia and London: W. B. Saunders & Co. 1902. Polished Buckram, $4.50 net; half Morocco, $5.50 net.

Students and practitioners will find this volume an invaluable guide to the diagnosis and treatment of fractures. The descriptions are given with a fullness of detail and are accompanied with a wealth of illustrations that cannot be found in any work on general surgery. Instead of dealing with theories, the student's attention is directed to actual conditions as they exist in fractured bones. Almost every detail in diagnosis and treatment is fully illustrated by original drawings and half-tone plates.

The chapters begin with brief but comprehensive reviews of the surgical anatomy of the parts under consideration. R.

